# Supplementary material for: Impact of commonly used drugs on the composition and metabolic function of the gut microbiota
Source: Nat Commun. 2020 Jan 17;11:362. doi: 10.1038/s41467-019-14177-z (PMC6969170; doi:10.1038/s41467-019-14177-z)
Supplement: Supplementary file 2 — Description of additional supplementary files [file 41467_2019_14177_MOESM2_ESM.docx]

File Name: Supplementary Data 1

Description: Summary descriptives of medication use general population, IBD and IBS cohorts

File Name: Supplementary Data 2

Description: Summary medication use characteristics: medication combinations

File Name: Supplementary Data 3

Description: Correlation coefficients for medication users in general population cohort

File Name: Supplementary Data 4

Description: Correlation coefficients for medication users in IBD cohort

File Name: Supplementary Data 5

Description: Correlation coefficients for medication users in IBS cohort

File Name: Supplementary Data 6

Description: Association between medication use and shannon index in general population, IBD and IBS cohort

File Name: Supplementary Data 7

Description: PERMANOVA (adonis). Phenotype impact on microbiota compositon (Bray Curtis Distances) in the general population, IBD and IBS cohorts

File Name: Supplementary Data 8

Description: Summary statistics of individual taxa, selected and coloured by statistically significant results of the univariate meta-analysis.

File Name: Supplementary Data 9

Description: Summary statistics of individual pathways, selected and coloured by statistically significant results of the univariate meta-analysis.

File Name: Supplementary Data 10

Description: Summary statistics of individual taxa, selected and coloured by statistically significant results of the multivariate meta-analysis.

File Name: Supplementary Data 11

Description: Summary statistics of individual pathways, selected and coloured by statistically significant results of the multivariate meta-analysis.

File Name: Supplementary Data 12

Description: Users of a subtype of drugs vs non-users of that drug group for drugs-microbiome associations identified in the multivariate analyses of taxa and pathways & dosage analyses

File Name: Supplementary Data 13

Description: Summary statistics taxa & pathways vs ACE inhibitors

File Name: Supplementary Data 14

Description: Summary statistics taxa & pathways vs alpha blockers

File Name: Supplementary Data 15

Description: Summary statistics taxa & pathways vs angiotensin II receptor antagonists

File Name: Supplementary Data 16

Description: Summary statistics taxa & pathways vs anti androgen contraceptives

File Name: Supplementary Data 17

Description: Summary statistics taxa & pathways vs anti epileptics

File Name: Supplementary Data 18

Description: Summary statistics taxa & pathways vs anti histamines

File Name: Supplementary Data 19

Description: Summary statistics taxa & pathways vs antibiotics

File Name: Supplementary Data 20

Description: Summary statistics taxa & pathways vs benzodiazepinez derivates

File Name: Supplementary Data 21

Description: Summary statistics taxa & pathways vs beta blockers

File Name: Supplementary Data 22

Description: Summary statistics taxa & pathways vs beta sympathomimetic inhalers

File Name: Supplementary Data 23

Description: Summary statistics taxa & pathways vs bisphosphonates

File Name: Supplementary Data 24

Description: Summary statistics taxa & pathways vs calcium channel blockers

File Name: Supplementary Data 25

Description: Summary statistics taxa & pathways vs calcium

File Name: Supplementary Data 26

Description: Summary statistics taxa & pathways vs laxatives

File Name: Supplementary Data 27

Description: Summary statistics taxa & pathways vs levothyroxine

File Name: Supplementary Data 28

Description: Summary statistics taxa & pathways vs metformin

File Name: Supplementary Data 29

Description: Summary statistics taxa & pathways vs NSAIDs

File Name: Supplementary Data 30

Description: Summary statistics taxa & pathways vs opiats

File Name: Supplementary Data 31

Description: Summary statistics taxa & pathways vs oral antidiabetics

File Name: Supplementary Data 32

Description: Summary statistics taxa & pathways vs oral contraceptives

File Name: Supplementary Data 33

Description: Summary statistics taxa & pathways vs oral steroids

File Name: Supplementary Data 34

Description: Summary statistics taxa & pathways vs other antidepressants

File Name: Supplementary Data 35

Description: Summary statistics taxa & pathways vs paracetamol

File Name: Supplementary Data 36

Description: Summary statistics taxa & pathways vs platelet aggregation inhibitors

File Name: Supplementary Data 37

Description: Summary statistics taxa & pathways vs proton pump inhibitors

File Name: Supplementary Data 38

Description: Summary statistics taxa & pathways vs SSRI antidepressants

File Name: Supplementary Data 39

Description: Summary statistics taxa & pathways vs statins

File Name: Supplementary Data 40

Description: Summary statistics taxa & pathways vs steroid inhaler

File Name: Supplementary Data 41

Description: Summary statistics taxa & pathways vs nasal steroids

File Name: Supplementary Data 42

Description: Summary statistics taxa & pathways vs thiazide diuretics

File Name: Supplementary Data 43

Description: Summary statistics taxa & pathways vs tricyclic antidepressants

File Name: Supplementary Data 44

Description: Summary statistics taxa & pathways vs triptans

File Name: Supplementary Data 45

Description: Summary statistics taxa & pathways vs vitamin D

File Name: Supplementary Data 46

Description: Summary statistics taxa & pathways vs vitamin K antagonists

File Name: Supplementary Data 47

Description: Summary statistics cohort specific taxonomy

File Name: Supplementary Data 48

Description: Summary statistics cohort specific pathways

File Name: Supplementary Data 49

Description: Summary statistics taxa & pathways vs IBD drugs: anti-TNFα, thiopurines and mesalazines

File Name: Supplementary Data 50

Description: Species level stratified abundances of pathways and gene families in PPIs

File Name: Supplementary Data 51

Description: Species level stratified abundances of pathways and gene families in metformin

File Name: Supplementary Data 52

Description: Species level stratified abundances of pathways and gene families in antibiotics

File Name: Supplementary Data 53

Description: Species level stratified abundances of pathways and gene families in laxatives

File Name: Supplementary Data 54

Description: Associations between total counts of antibiotic resistance markers and medication use

File Name: Supplementary Data 55

Description: Associations between individual antibiotic resistance markers and medication use

File Name: Supplementary Data 56

Description: Annotation of pathways
