# Supplementary material for: Impact of commonly used drugs on the composition and metabolic function of the gut microbiota
Source: Nat Commun. 2020 Jan 17;11:362. doi: 10.1038/s41467-019-14177-z (PMC6969170; doi:10.1038/s41467-019-14177-z)
Supplement: Supplementary file 59 — Reporting Summary [file 41467_2019_14177_MOESM59_ESM.pdf]

nature research

Corresponding author(s): R.K.Weersma

Last updated by author(s): Nov 19, 2019

Reporting Summary

Nature Research wishes to improve the reproducibility of the work that we publish. This form provides structure for consistency and transparency in reporting. For further information on Nature Research policies, see Authors & Referees and the Editorial Policy Checklist .

Statistics

For all statistical analyses, confirm that the following items are present in the figure legend, table legend, main text, or Methods section.

n/a	Confirmed	
☐	☒	The exact sample size (*n*) for each experimental group/condition, given as a discrete number and unit of measurement
☐	☒	A statement on whether measurements were taken from distinct samples or whether the same sample was measured repeatedly
☐	☒	The statistical test(s) used AND whether they are one- or two-sided<br>*Only common tests should be described solely by name; describe more complex techniques in the Methods section.*
☐	☒	A description of all covariates tested
☐	☒	A description of any assumptions or corrections, such as tests of normality and adjustment for multiple comparisons
☐	☒	A full description of the statistical parameters including central tendency (e.g. means) or other basic estimates (e.g. regression coefficient) AND variation (e.g. standard deviation) or associated estimates of uncertainty (e.g. confidence intervals)
☐	☒	For null hypothesis testing, the test statistic (e.g. *F*, *t*, *r*) with confidence intervals, effect sizes, degrees of freedom and *P* value noted<br>*Give P values as exact values whenever suitable.*
☒	☐	For Bayesian analysis, information on the choice of priors and Markov chain Monte Carlo settings
☒	☐	For hierarchical and complex designs, identification of the appropriate level for tests and full reporting of outcomes
☐	☒	Estimates of effect sizes (e.g. Cohen's *d*, Pearson's *r*), indicating how they were calculated

Our web collection on statistics for biologists contains articles on many of the points above.

Software and code

Policy information about availability of computer code

Data collection: No software was used for data collection

Data analysis:
kneadData v0.4.6.1
MetaPhlAn2 v 2.2,
HUMAnN2 v 0.10.0,
ShortBRED v 1.0,
R v 3.3.2
metagen (R package) v. 4.8-4
vegan (R package) v. 2.4-1
Custom code was used for data analysis. Code is publicly available at:
https://github.com/GRONINGEN-MICROBIOME-CENTRE/Groningen-Microbiome/blob/master/Projects/Medication_metanalysis/Analysis_steps.md

For manuscripts utilizing custom algorithms or software that are central to the research but not yet described in published literature, software must be made available to editors/reviewers. We strongly encourage code deposition in a community repository (e.g. GitHub). See the Nature Research guidelines for submitting code & software for further information.

Data

Policy information about availability of data

All manuscripts must include a data availability statement. This statement should provide the following information, where applicable:

- Accession codes, unique identifiers, or web links for publicly available datasets
- A list of figures that have associated raw data
- A description of any restrictions on data availability

The raw metagenomics sequencing reads are available for all three cohorts under request in the European Genome-phenome Archive (EGA: https://ega-

archive.org). The accession number of the 1000IBD cohort is EGAD00001004194, of the LifeLinesDEEP cohort is EGAD00001001991 and for the MIBS is EGAD00001002668.

Field-specific reporting

Please select the one below that is the best fit for your research. If you are not sure, read the appropriate sections before making your selection.

- [x] Life sciences
- [ ] Behavioural & social sciences
- [ ] Ecological, evolutionary & environmental sciences

For a reference copy of the document with all sections, see nature.com/documents/nr-reporting-summary-flat.pdf

Life sciences study design

All studies must disclose on these points even when the disclosure is negative.

Sample size	All metagenomic samples (n=1883) from three different cohorts with an extensive description on medication use per each participant at the time of sampling were selected.
Data exclusions	In the IBD cohort, 67 patients with stoma, pouches or short bowel syndrome were excluded. Furthermore, samples with a sequencing depth < 10 million reads were removed (n=30, 22 samples from the IBD cohort and 8 samples from the Maastricht IBS cohort).
Replication	Each cohort was analyzed individually and results were combined in a meta-analysis.
Randomization	No randomization was done
Blinding	This study is observational therefore no blinding was performed.

Reporting for specific materials, systems and methods

We require information from authors about some types of materials, experimental systems and methods used in many studies. Here, indicate whether each material, system or method listed is relevant to your study. If you are not sure if a list item applies to your research, read the appropriate section before selecting a response.

Materials & experimental systems

n/a	Involved in the study	
x		Antibodies
x		Eukaryotic cell lines
x		Palaeontology
x		Animals and other organisms
	x	Human research participants
x		Clinical data

Methods

n/a	Involved in the study	
x		ChIP-seq
x		Flow cytometry
x		MRI-based neuroimaging

Human research participants

Policy information about studies involving human research participants

Population characteristics	For this study we used three independent Dutch cohorts:<br>1) a general population cohort, LifeLines-DEEP, consisting of 1539 individuals (mean age 44.8, SD: 13.7, 58% females, mean BMI =25.3, SD=4.2)<br>2) 544 patients with IBD from the 1000IBD cohort of the University Medical Center of Groningen (UMCG) (mean age 42.8, SD=14.8, 59% females, BMI=22.5 SD=5)<br>3) an IBS case–control cohort with 313 participants from Maastricht University Medical Center+ (MUMC+) (mean age 45.4, SD=17.7, 65% females, BMI=24.6 SD=4)
Recruitment	Participants volunteer in the sample collection. Informed consent forms were available for all participants and all were 18 years or older at time of faecal sampling. No selection criteria was applied in the population cohort, participants from the clinical cohorts were recruited based on their clinical phenotype (inflammatory bowel disease or irritable bowel syndrome)
Ethics oversight	Institutional ethics review board (IRB) approval was available for all three cohorts. Both the Lifelines DEEP and UMCG IBD cohort were approved by the UMCG IRB (ref. M12.113965 and IRB-number 2008.338, respectively). The Maastricht IBS cohort was approved by the MUMC+ IRB (ref. MEC 08-2-066.7/pl).

Note that full information on the approval of the study protocol must also be provided in the manuscript.
